# Association of Dietary Factors With Grip Strength, Body Fat, and Prevalence of Sarcopenic Obesity in Rural Korean Elderly With Cardiometabolic Multimorbidity

**DOI:** 10.3389/fnut.2022.910481

**Published:** 2022-07-14

**Authors:** Jieun Kim, Younghwa Baek, Kyoungsik Jeong, Siwoo Lee

**Affiliations:** Division of Korean Medicine Data, Korea Institute of Oriental Medicine, Daejeon, South Korea

**Keywords:** diet, elderly, macronutrients, sarcopenic obesity, cardiometabolic multimorbidity

## Abstract

**Background and Aims:**

Aging accompanied by cardiometabolic multimorbidity (CM) promotes chronic low-grade inflammation, increased oxidative stress, and insulin resistance (IR), which result in loss of muscle mass and functional impairment. Better quality diets have been directly associated with muscle health and decreased risk of all-cause mortality. However, no study has investigated the relationship of dietary factors with grip strength, body composition, and prevalence of sarcopenic obesity (SO) in Korean rural residents according to their CM pattern. Therefore, we aimed to examine this association among this population.

**Materials and Methods:**

This cross-sectional study utilized data from 932 rural residents aged ≥ 65 years. An exploratory tetrachoric factor analysis revealed four multimorbidity patterns: CM, inflammatory disease, respiratory disease, and cancer and other diseases. All participants were categorized into the CM and non-CM groups. Skeletal muscle mass and the prevalence of sarcopenia were estimated using bioelectrical impedance analysis (BIA). Dietary assessment was analyzed using a validated 106-item food frequency questionnaire. Adjusted multiple linear regression and multivariate logistic regression were employed to examine the association of dietary factors with muscle strength, quality, and SO prevalence ratio in elderly participants.

**Results:**

The mean age of the participants was 71.8 ± 0.1 years (65.8% women). Dietary fat and protein intake were positively correlated with handgrip strength in women with CM, after adjusting for covariates (*p* = 0.001). Similarly, protein intake (g/kg) was positively associated with appendicular skeletal muscle mass (ASM; kg/m^2^) and ASM (%) in both sexes in the CM and non-CM groups. Regarding the tertiles of wheat intake (g/d), 2.1-fold increase in SO prevalence ratios [prevalence ratio (PR): 2.149, confidence intervals (CIs): 1.134–4.071] was observed in the highest tertile (T3: 269.1 g/d), compared to the lowest tertile (Q1: 8.6 g/d) in the CM group. Higher tertile of meat intake (T2: 34.8 g/d, T3: 99.5 g/d) had a 2-fold increase in SO (PR: 1.932, CIs: 1.066–3.500) compared to the lowest tertile (T1: 9.2 g/d) in the CM group.

**Conclusion:**

Overconsumption of wheat and meat negatively impacted the development of SO, while protein intake was positively associated with grip strength and skeletal muscle mass in elderly Koreans with CM.

## Introduction

Sarcopenic obesity (SO) is related to not only aging but also cardiovascular diseases (CVDs) and metabolic diseases such as insulin resistance (IR), type 2 diabetes (T2DM), and obesity (1). A recent review reported that more than one in 10 older adults face a health crisis due to SO (2).

Cardiometabolic multimorbidity (CM), i.e., the presence of two or more chronic diseases such as T2DM and CVDs, requires comprehensive care to prevent functional incapacity and worsening in the elderly population (3, 4). Compared to patients with single diseases, those with multimorbidity have cumulative interactions between diseases (5). Aging increases susceptibility to multimorbidity due to multisystem homeostatic dysregulation (3). A report from 18 countries identified a 4–8 times greater risk of all-cause mortality in participants with more than three chronic diseases when compared to those with a single disease (6). Physiological, hormonal, and other lifestyle factors may act as mediators between CM and SO (7–9). In geriatrics, multimorbidity is a more common clinical problem that affects several pathways (3). Therefore, multidisciplinary integrated care approaches are required so as to not only focus on single diseases or risk factors.

A recent study pointed out the requirement of muscle functional evaluation in the sarcopenia research field (10), particularly, related to the coexistence of sarcopenia and obesity, due to accelerated loss of muscle mass, strength, and quality in the elderly (11). Handgrip strength has been reported as a strong predictor of all-cause and CVD mortality in people with diverse economic and sociocultural backgrounds in a large longitudinal prospective study (12). Decreased handgrip strength has also been reported in adults (aged > 50 years) with multimorbidity compared to those without chronic diseases (13).

Adequate nutrition is an essential contributor to maintaining health and decreasing the risk of all-cause mortality, CVDs, and T2DM in older adults (14–16). Macronutrients (carbohydrates, fat, and proteins) play an important role in preventing muscle loss and IR in aging muscle biology (17–19). A healthy and balanced diet rich in vegetables, fruits, legumes, and fish is associated with a lower 10-year risk of incidence of CVDs than the typical white rice and grain-based Korean diet in elderly people (20). The recent Korean National Health and Nutrition Examinations Survey (KNHANES) reported that excessive carbohydrate, low fruit, and imbalanced food and nutrient intakes contribute to cardiometabolic abnormalities in rural residents (21). However, no study has investigated that associations between nutritional risk factors and grip strength, body composition, and prevalence of SO by considering regional specificity in Korean rural residents. We hypothesized that elderly with CM would have an inadequate dietary intake compared to the non-cardiometabolic multimorbidity pattern (CMP) group. Thus, this study aimed to identify CM patterns in elderly Koreans living in a rural area and examine how the dietary factors, grip strength, body composition, and prevalence of SO among this population may relate to their CM pattern.

## Materials and Methods

### Design, Setting, and Participants

Data were collected from secondary research conducted in February 2014 to assess health status (22, 23), Korean Medicine (KM) constitutional type (24), and health-related clinical outcomes of the general population according to sub-health status. Baseline survey data were obtained after the participants provided written informed consent. Each participant was asked to visit the local health examination center for a health survey and examination. To use well-curated validation datasets for the present analysis, approvals were obtained from the web-based Korean Medicine Data Center (KDC) electronic data capture system by the KDC of the Korean Institute of Oriental Medicine (25).

Anthropometric measurements [height, weight, waist circumference, systolic blood pressure (BP), diastolic BP, and handgrip strength] and laboratory data [triglycerides (TG), high-density lipoprotein cholesterol (HDL-C), and fasting plasma glucose after overnight fasting] were recorded. Of the 1,890 participants in the survey data, 932 rural residents in Gyeongju, South Korea, aged ≥65 years, were included in the present analysis.

### Definition of Cardiometabolic Multimorbidity Pattern

We defined multimorbidity (3, 4) as the co-occurrence of any of the following 27 chronic diseases: stroke, transient ischemic attack, angina (or myocardial infarction), hypertension, dyslipidemia, pulmonary tuberculosis, thyroid disorder (other than thyroid cancer), chronic gastritis, ulcer (gastric/duodenal), diabetes, intestinal polyposis syndrome, acute hepatic disease, fatty liver, chronic hepatitis (or hepatic cirrhosis), cholelithiasis (or cholecystitis), chronic bronchitis, asthma, allergy, arthritis, cystitis, cataract, glaucoma, depression, Parkinson’s disease, osteoporosis, prostatic hyperplasia, and cancer. A combination of methods is used to determine the presence of multimorbidity, including physician diagnosis and/or current use of medications for a particular disease (25–27).

Four multimorbidity patterns were identified among the participants: isolated CM [23.8% (*n* = 222)], respiratory diseases [12.4% (*n* = 116)], inflammatory diseases [12.3% (*n* = 115)], and cancer and other diseases [19.6% (*n* = 183)] (data not shown). A total of 4.7% of the participants had all four patterns, whereas 26.2% (*n* = 244) had none of the four patterns. CM pattern included diabetes, dyslipidemia, hypertension, and angina (or myocardial infarction). Respiratory disease pattern included pulmonary tuberculosis, chronic bronchitis, asthma, and allergies. Inflammatory diseases pattern included chronic gastritis, arthritis, cystitis, cataracts, and osteoporosis. Finally, cancer and other diseases pattern included acute hepatic disease, fatty liver, chronic hepatitis, glaucoma, depression, and cancers. Participants with cardiometabolic multimorbidity, such as diabetes, dyslipidemia, hypertension, and angina (or myocardial infarction), were extracted and further categorized into a CM pattern only group (50.0%, *n* = 466; if not, non-CMP).

### Definition of Sarcopenic Obesity

Sarcopenic obesity is generally defined as the coexistence of sarcopenia and obesity (11). Recently, broad diagnostic criteria have been employed to identify both sarcopenia and obesity, such as sex-specific weak handgrip strength, body mass index (BMI), or total body fat percentage (%BF) (26). We used the cross-validated equation (27) for the estimation of appendicular skeletal muscle mass (ASM) of the four limbs using bioelectrical impedance analysis (BIA; InBody 720, Seoul, Korea). Muscle strength was assessed by handgrip strength (kg) on either or both hands using a grip strength dynamometer (TANITA 6103, Tokyo, Japan); the maximum reading of two trials was used.

#### Sarcopenia

##### Muscle Mass (Kg, %)

We defined sarcopenia as having low ASM in kilograms (kg), as reflected by:

**A. ASM (kg/m^2^):** Skeletal muscle mass index was calculated as the sum of the ASM divided by the square of height (kg/m^2^). Sarcopenia was defined as a skeletal muscle mass index <7.0kg/m^2^ for men and <5.7 kg/m^2^ for women using BIA, based on the Asian Working Group for Sarcopenia (AWGS) 2019 criteria (28).

**B. ASM (%):** ASM was calculated as a percentage of body weight according to Janssen’s formula (ASM/weight [kg] × 100 [%]) and as one standard deviation (SD) below the value of a young reference group (men = 51, women = 128, aged 20–40 years) (29).

##### Muscle Strength (Kg)

Following the AWGS 2019 recommendation, a handgrip strength < 28.0 kg for men and < 18.0 kg for women or the lowest quintile of muscle strength among the study participants indicated low muscle strength (28).

#### Obesity

Obesity was defined as BMI ≥ 25 kg/m^2^ (30) or the upper two quintiles for%BF using BIA for each sex. For women, %BF quintiles were Q1: 28.9, Q2: 33.2–36.4, Q3: 36.5–39.3, Q4: 39.4–42.0, and Q5: 42.1–51.7, and for men, these values were Q1: 18.8, Q2: 22.1–26.0, Q3: 26.1–29.1, Q4: 29.2–32.8, and Q5: 39.4–42.0.

Using these cut-offs, we identified two subgroups: SO**A** and SO**B**.

### Dietary Assessment

Dietary factors were assessed using a validated quantitative food frequency questionnaire, which contained 106 food items with serving sizes based on the Korean Genome and Epidemiology Study (31). Daily food amount in grams and frequency were calculated from the energy (kcal/d), protein, calcium, phosphorus, iron, vitamin B1, vitamin B2, niacin, vitamin C, zinc, vitamin B6, potassium, sodium, vitamin E, and fiber based on the recommended nutritional intake from the Dietary Reference Intakes for Koreans (KDRIs) [Korean Nutrients Society (32)] to evaluate the nutrient adequacy of the individual diet among the elderly participants. Foods were classified into carbohydrate-rich (white rice, mixed rice, noodles, and wheat) or protein-rich (beans, nuts, tofu, fish, meat, and poultry eggs) group based on the Korean Nutrient Database.

### Outcome Variables

The primary outcome variable was the association of macronutrient intake with handgrip strength and body composition of the participants by CM pattern. The secondary outcome variable was the association between food sources and the prevalence of SO in the elderly participants by CM pattern.

### Covariates

Participants’ age, sex, BMI, health-related behaviors (smoking status, alcohol consumption, and physical activity level), working hours, KM type, and energy intake (kcal/day) were used as covariates in the statistical analysis. Previously, we confirmed high-risk associations with cardiometabolic outcomes (33) and inflammatory status (34) based on the two KM types in Korean adults. Age, BMI, and energy intake were used as continuous variables, and sex (male vs. female), smoking (no vs. yes), alcohol consumption (no vs. yes), physical activity (no vs. yes), working hours (≤8 h/day or ≤20 h/week vs. >8 h/day or >20 h/week), and KM type (Taeeum vs. Non-Taeeum) were used as categorical variables. Active daily working hours and KM type were also included as strong predictors to build better adjustment for baseline data. Both variable types showed considerable regional (rural area) or national features.

### Ethics Statement

This study was approved by the Korean Institute of Oriental Medicine Ethics Committee (No. I-1401/001-001-01) and the Ethics Committee of the Institute of Medicine at the Seoul National University (IRB No. 1310-060-528). Written informed consent was obtained from all participants.

### Statistical Analysis

We followed a previously published method to identify non-random multimorbid groups. Multimorbidity patterns were analyzed using exploratory factor analysis (35), which identifies the tendencies of diseases to co-occur, by selecting sets of variables with potentially common underlying causal factors. A tetrachoric correlation matrix will lead to more valid results for the assessment of the correlation structure between the variables to account for binary morbidity data (36). The number of factors extracted by the scree plot was utilized, in which the eigenvalues of the correlation matrix were represented in descending order to produce the inflection point of the curve, an eigenvalue of 1.0. To facilitate interpretation, the factors were rotated using the oblique rotation (oblimin) method. The Kaiser–Meyer–Olkin method was implemented to determine the adequacy of the sample in the factor analysis. To determine the most appropriate multimorbidity pattern, we selected variables with factor loadings ≥ 0.30 (37, 38) and classified them into patterns based on common disease features (Supplementary Table 1).

Frequencies and percentages were used for categorical variables in the descriptive analysis. Chi-square (χ^2^) test was used to compare general and health-related characteristics (sex, smoking, drinking, physical activity, working hours, and KM type). All data on continuous variables related to muscle strength (handgrip strength and weight-adjusted handgrip strength), body composition, sarcopenia (ASM, kg, kg/m^2^, and %), and cardiometabolic profiles are presented in Table 1 and nutrients and food groups in Table 2. *P* values from the multiple comparisons were obtained using a Bonferroni corrected one-way analysis of variance and analysis of covariance (ANCOVA). Multiple linear regression models were employed to determine the best risk predictors between handgrip strength (muscle strength, kg), weight-adjusted handgrip strength (%), body fat mass (kg, %), ASM (kg/m^2^), and ASM (%). Dietary food sources (g), handgrip strength (kg), and body composition [body fat mass (%), and ASM (kg/m^2^, %)] variables were assessed for normality using the Shapiro–Wilk test. Correlations of dietary food sources (g) with handgrip strength (kg) and body composition (kg/m^2^, %) were determined using Pearson correlation coefficient. Multivariable logistic regression was used to evaluate the association between macronutrient consumption (%) and prevalence of SO according to the CM pattern; adjusted prevalence ratios (PRs) and 95% confidence intervals (CIs) were also estimated. All analyses were performed using SAS version 9.4 (SAS Institute Inc., Cary, NC, United States). All statistical tests were two-tailed, and *p*-values < 0.05 were considered statistically significant.

**TABLE 1:** General characteristics, handgrip strength, body composition and cardiometabolic profiles of the participants according to their CM pattern^1^.

Multimorbidity pattern	All (*n* = 932)	Non-CM (*n* = 466)	CM (*n* = 466)	*P-value*
**Characteristics**				
Age, years (mean ± SE)	71.8 ± 0.1	71.0 ± 0.2	72.0 ± 0.2	0.062
Sex (*n*, %)				
Men	319(34.2)	172(36.9)	147(31.6)	0.084
Women	613(65.8)	294(63.1)	319(68.5)	
**Smoking (%)**				
No	657(70.5)	319(68.5)	338(72.5)	0.172
Yes	275(29.5)	147(31.5)	121(27.5)	
**Drinking (%)**				
No	413(44.3)	215(46.1)	198(42.5)	0.262
Yes	519(55.7)	251(53.9)	268(57.5)	
**Physical activity (%)**				
No	770(82.6)	391(83.9)	379(81.3)	0.300
Yes	162(17.4)	75(16.1)	87(18.7)	
**Working hours (%)**				
≤8 h/day or ≤ 20 h/week	324(34.8)	128(27.5)	196(42.1)	<0.0001
>8 h/day or > 20 h/week	608(65.2)	338(72.5)	270(57.9)	
**KM type^2^ (%)**				
Non-Taeeum	617(66.2)	347(74.5)	270(57.9)	**<0.0001**
Taeeum	315(33.8)	119(25.5)	196(42.1)	
**Hand grip strength (kg)**				
*Men*		27.8 ± 0.7	28.9 ± 0.8	0.296
*Women*		15.3 ± 0.3	15.6 ± 0.3	0.493
**HGSWR^3^ (%)**				
*Men*		43.9 ± 1.1	41.6 ± 1.2	0.151
*Women*		27.9 ± 0.6	26.7 ± 0.6	0.153
**Body composition (mean ± SE)**				
BMI, kg/m**^2^**		24.0 ± 0.1	**26.0** ± **0.1**	**<0.0001**
Body fat mass, kg		18.7 ± 0.3	**22.9** ± **0.3**	**<0.0001**
Body fat mass, %		32.0 ± 0.3	**36.2** ± **0.3**	**<0.0001**
**ASM^4^ (kg/m^2^)**				
*Men*		7.3 ± 0.0	**7.4** ± **0.0**	**<0.0001**
*Women*		6.4 ± 0.0	**6.5** ± **0.0**	**<0.0001**
**ASM^5^ (%)**				
*Men*		**31.4** ± **0.2**	29.1 ± 0.2	**<0.0001**
*Women*		**26.7** ± **0.1**	25.2 ± 0.1	**<0.0001**
**Sarcopenic Obesity^6^ (%)**				
*height-adjusted^4^ (women only)*	70(7.5)	14(3.0)	56(12.0)	**<0.0001**
*weight-adjusted^5^*	155(33.3)	31(6.6)	124(30.5)	**<0.0001**
**Cardio-metabolic profiles (mean ± SE)**		1.8 ± 0.1	**2.2** ± **0.1**	**<0.0001**
WC, cm		80.4 ± 0.4	**82.2** ± **0.4**	**0.000**
Systolic BP, mmHg		136.1 ± 0.6	**139.2** ± **0.7**	**0.001**
Diastolic BP, mmHg		76.5 ± 0.4	**77.8** ± **0.4**	**0.048**
Fasting plasma glucose, mg/dL		95.1 ± 1.1	**106.3** ± **1.1**	**<0.0001**
HDL-C, mg/dL		**53.1** ± **0.6**	51.1 ± 0.6	**0.018**
TG, mg/dL		123.8 ± 3.5	**149.9** ± **3.5**	**<0.0001**

*^1^Cardio-metabolic multimorbidity (CM) pattern included diabetes, dyslipidemia, and hypertension, and angina (or myocardial infarction).*

*^2^Korean medicine (KM) type was diagnosed into Taeeum or non-Taeeum type according to participants’ personal and physiological character by KM doctors.*

*^3^Hand grip strength weight ratio was calculated by hand grip strength/weight x 100.*

*^4^ASM, kg/m^2^ by Asian Working Group for Sarcopenia (AWGS) 2019 criteria.*

*^5^ASM, % by Janssen’s formula [ASM/weight (kg) × 100 (%)].*

*^6^Sarcopenic Obesity (SO) is generally defined as the coexistence of sarcopenia and obesity.*

*P-values were obtained from Rao-Scott chi-square tests for categorical variables and Bonferroni multiple comparison of one-way analysis of variance and analysis of covariance (ANCOVA).*

*Least-square means ± SE adjusted for age and sex. Bold letters represent a significant value.*

**TABLE 2:** Daily energy and nutrients intakes and food groups of the participants according to their CM pattern.

Dietary factors	Non-CM (*n* = 466)	CM (*n* = 466)	*P*-value
**Energy (kcal/d)^1^**			
% RDA	85.1 ± 1.1	84.5 ± 1.0	0.708
Men	1625.6 ± 31.7	1674.0 ± 34.3	0.302
Women	1433.3 ± 23.1	1399.5 ± 22.1	0.291
**Nutrients^2^**			
Carbohydrates (g)	278.7 ± 1.0	279.0 ± 1.0	0.826
Fat (g)	18.4 ± 0.3	18.0 ± 0.3	0.356
Protein (g)	46.7 ± 0.4	47.2 ± 0.4	0.361
Protein [g, per body weight (kg)]	**0.81**±**0.0**	0.76 ± 0.0	**<0.0001**
C:F: P (%)	74.8: 10.8: 12.4	75.1: 10.4: 12.5	N/S
Vitamin A, μgRAE	306.5 ± 8.8	317.4 ± 8.8	0.384
Vitamin B1, mg	0.7 ± 0.0	0.7 ± 0.0	0.978
Vitamin B2, mg	0.6 ± 0.0	0.6 ± 0.0	0.734
Niacin	11.5 ± 0.1	11.5 ± 0.1	0.912
Vitamin C, mg	69.9 ± 1.8	72.0 ± 1.8	0.419
Vitamin E, mg	5.6 ± 0.1	5.6 ± 0.1	0.908
Vitamin B6, mg	1.2 ± 0.0	1.2 ± 0.0	0.856
Potassium, mg	1714.4 ± 25.5	1746.2 ± 25.5	0.378
Calcium, mg	321.7 ± 6.8	328.6 ± 6.8	0.477
Phosphorus, mg	735.6 ± 6.1	747.7 ± 6.1	0.161
Iron, mg	7.7 ± 0.1	7.9 ± 0.1	0.137
Fiber, g	4.6 ± 0.1	4.8 ± 0.1	0.065
Sodium, mg	2420.9 ± 57.4	2450.3 ± 57.4	0.717
**Food groups (g/d)^2^^,^ ^3^**			
*Carbohydrates-rich*			
White rice	648.7 ± 10.0	635.3 ± 13.0	0.416
Mixed rice	607.8 ± 7.9	613.8 ± 6.9	0.574
Noodles	32.0 ± 1.7	30.0 ± 1.7	0.403
Wheats^4^	46.0 ± 1.9	43.4 ± 1.9	0.345
***Protein-rich***			
Beans-nuts-tofu	38.3 ± 2.3	43.1 ± 2.3	0.141
Fish	23.3 ± 1.0	25.1 ± 1.0	0.237
Meats	24.0 ± 1.0	21.5 ± 1.0	0.072
Poultry-eggs	9.2 ± 0.5	9.4 ± 0.6	0.819

*^1^Adjusted age only.*

*^2^Least-square means ± SE adjusted for age, sex and energy intake (kcal).*

*^3^Food groups were surveyed using short-form of the food frequency questionnaires (FFQ) which includes grains (white rice, mixed rice, noodles and wheats, potatoes and sweet potatoes).*

*^4^Wheats: wheat and other types of refined grains based extra food intakes for snacks; cereals, breads, ricecakes.*

*P-values were obtained from Bonferroni multiple comparison of one-way analysis of variance and analysis of covariance (ANCOVA). Bold letters represent a significant value.*

## Results

General characteristics, handgrip strength, body composition, and cardiometabolic profiles of the participants according to the CM pattern are presented in Table 1. The mean age of participants was 71.8 ± 0.1 years, and 65.8% (*n* = 613) were women. No significant health-related behaviors were observed in the CM and non-CM groups. In total, 72.5% of those in the non-CM group worked for longer durations (>8 h/day or > 20 h/week) compared to those in the CM group (*p* < 0.0001). Regarding the KM type, 17.6% more Taeeum-type individuals were observed in the CM group than in the non-CM group (*p* < 0.0001).

Regarding handgrip strength (kg) and weight-adjusted handgrip strength (%), no significant differences were found between the CM and non-CM groups. Body composition showed that BMI (kg/m^2^) and body fat mass in kilogram (kg) and in percentage (%; non-CM: 32.0 ± 0.3 vs. CM: 36.2 ± 0.3) were significantly higher in the CM group than in the non-CM group (*p* < 0.0001). Both men (non-CM: 7.3 ± 0.0 vs. CM: 7.4 ± 0.0, *p* < 0.0001) and women (non-CM: 6.4 ± 0.0 vs. CM: 6.5 ± 0.0, *p* < 0.0001) had higher height-adjusted ASM (kg/m^2^) in the CM group than in the non-CM group. However, weight-adjusted ASM (%) in both sexes was significantly lower in the CM group than in the non-CM group (*p* < 0.0001). The prevalence of SO was 7.5% [height-adjusted, *n* = 70 (women only)] and 33.3% (weight-adjusted, *n* = 155), respectively. Height-adjusted SO was observed only in women. Significant group differences in SO prevalence were observed by CM pattern. Regarding the cardiometabolic profiles, higher waist circumference (cm), systolic and diastolic BP (mmHg), fasting plasma glucose (mg/dl), and TG (mg/dl) and lower HDL-C (mg/dl) were observed in the CM group than in the non-CM group (*p* < 0.05; Table 1).

Table 2 shows the daily energy (kcal/d), nutrient, and food groups (g/d) consumed by the participants according to their CM patterns. Overall, there were no significant differences in the daily energy intake of the participants (both sexes) between the groups. Regarding macronutrient intake, protein intake (g) was higher (non-CM: 46.1 ± 0.4 vs. CM: 47.6 ± 0.4, *p* = 0.009) and protein distribution (g/kg) was lower (non-CM: 0.81 ± 0.0 vs. CM: 0.76 ± 0.0, *p* < 0.0001) in the CM group than in the non-CM group. Regarding micronutrient intake, the vitamin B1, B2, potassium, phosphorus, and iron levels were higher in the CM group than in the non-CM group (*p* < 0.05). No significant differences were observed in the food groups between the two groups (Table 2).

The association between macronutrient intake, handgrip strength, and body composition of the participants according to their CM patterns is presented in Table 3. Regarding carbohydrate intake (g), there was a negative relationship between handgrip strength (kg: beta = −0.05, *p* = 0.001 and *^weight–adjusted^*%: −0.09, *p* = 0.001, women only) and body fat mass (kg: beta = −0.01, *p* = 0.046 and %: beta = −0.02, *p* = 0.017, women only) in the CM group. Meanwhile, fat intake was positively associated with body fat mass (% only) in women in the CM group. Dietary fat and protein intake (g) [g per body weight (g/kg)] were positively related to handgrip strength (both kg and *^weight–adjusted^*%) in women in the CM group after adjusting for covariates (*p* = 0.001). Similarly, protein intake (g/kg) showed significantly positive associations with ASM (kg/m^2^) and ASM (%) in both sexes in the CM and non-CM groups (Table 3).

**TABLE 3:** The association between macronutrients intake, handgrip strength, and body composition of the participants according to their CM pattern.

Macronutrients intake	Carbohydrates (g)				Fat (g)				Protein (g)				Protein [g, per body weight (kg)]			
	Non-CM		CM		Non-CM		CM		Non-CM		CM		Non-CM		CM	
								
Variables	^*adjusted*^ *beta*	*P-value*	^*adjusted*^ *beta*	*P-value*	^*adjusted*^ *beta*	*P-value*	^*adjusted*^ *beta*	*P-value*	^*adjusted*^ *beta*	*P-value*	^*adjusted*^ *beta*	*P-value*	^*adjusted*^ *beta*	*P-value*	^*adjusted*^ *beta*	*P-value*
**Hand grip strength (kg)**																
*Men*	–0.019	0.623	0.008	0.743	0.040	0.707	–0.005	0.941	0.032	0.724	–0.009	0.885	–2.242	0.689	–4.147	0.327
*Women*	0.025	0.216	–0.050	0.001***	–0.094	0.100	0.157	0.001***	–0.035	0.496	0.120	0.001***	–2.092	0.446	3.811	0.039*
**HGSWR (%)**																
*Men*	–0.026	0.654	0.018	0.631	0.052	0.746	–0.012	0.911	0.035	0.802	–0.042	0.672	3.125	0.714	–0.342	0.957
*Women*	0.058	0.111	–0.088	0.001***	–0.212	0.040*	0.281	0.001***	–0.083	0.366	0.212	0.001***	2.585	0.602	10.577	0.001***
*Body composition*																
**Body fat mass, kg**																
*Men*	–0.006	0.574	–0.016	0.096	0.012	0.577	0.038	0.177	0.021	0.412	0.039	0.121	–1.519	0.326	0.061	0.971
*Women*	–0.007	0.380	–0.012	0.046*	0.038	0.177	0.030	0.088	0.015	0.442	0.023	0.100	–2.469	0.017*	–1.443	0.04*
**Body fat mass, %**																
*Men*	–0.011	0.461	–0.024	0.069	0.023	0.563	0.061	0.115	0.023	0.497	0.055	0.112	1.399	0.503	5.174	0.053
*Women*	0.010	0.424	–0.020	0.017*	–0.034	0.327	0.054	0.031*	–0.028	0.358	0.032	0.103	0.216	0.897	1.881	0.059
ASM, kg/m^2a^																
*Men*	0.000	0.734	0.000	0.799	0.000	0.763	0.000	0.975	0.000	0.819	–0.001	0.511	0.291	<0.0001***	0.282	<0.0001***
*Women*	0.001	0.026*	0.000	0.941	–0.001	0.037*	0.000	0.912	–0.001	0.040*	0.000	0.839	0.157	<0.0001***	0.116	<0.0001***
**ASM, %** *^b^*																
*Men*	–0.003	0.364	0.001	0.479	0.008	0.230	0.006	0.432	0.001	0.858	–0.007	0.166	1.611	<0.0001***	0.901	0.009**
*Women*	0.002	0.324	0.002	0.241	–0.005	0.290	–0.005	0.314	–0.004	0.412	–0.002	0.522	1.083	<0.0001***	0.580	0.001***

*Multiple linear regression was used to estimate after adjusting age, sex, BMI, energy intake(kcal), smoking, alcohol consumption, physical activity, working hours, and KM type.*

*^a^ASM, kg/m^2^ by Asian Working Group for Sarcopenia (AWGS) 2019 criteria.*

*^b^ASM, % by Janssen’s formula [ASM/weight (kg) × 100 (%)].*

Statistical significance was accepted at *P < 0.05, **P < 0.01, and ***P < 0.001. Bold letters represent a significant value.

**FIGURE 1:**
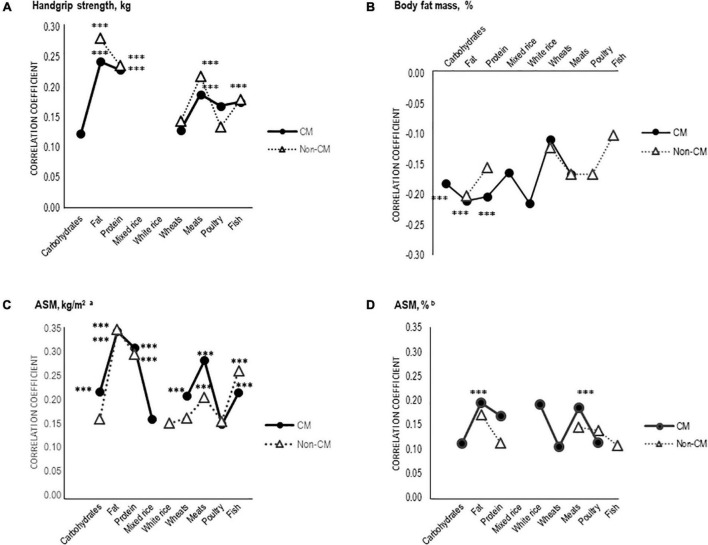
Relationship between handgrip strength, body fat mass, appendicular skeletal muscle mass (ASM), and dietary food sources (g) of the participants by CMP. Partial Pearson correlation coefficients controlling for individual energy intake were used for the correlation analysis. Statistical significance was accepted at ***P < 0.001. C. ASM, kg/m^2^ by Asian Working Group for Sarcopenia (AWGS) 2019 criteria. D. ASM, % by Janssen’s formula (ASM/weight [kg] × 100 [%]).

The correlations between dietary food sources (g), handgrip strength, and body composition of the participants are shown in Figure 1. As shown in *panel A*, handgrip strength positively correlated with dietary fat (non-CM: *r* = 0.28, CM: *r* = 0.24), protein (non-CM: *r* = 0.24, CM: *r* = 0.23), and meat (non-CM: *r* = 0.22, CM: *r* = 0.19) in the CM and non-CM groups (*p* < 0.001). In contrast, macronutrient intake was inversely related to body fat mass (%) in the CM group only (*p* < 0.001; *panel B*). Similar to the handgrip strength (*panel A)*, *panel C* reveals positive correlations between ASM (kg/m^2^) and macronutrients, including carbohydrate-rich wheat, meat, and fish intake, in the non-CM and CM groups (*p* < 0.001). However, ASM (kg/m^2^) showed a strong positive correlation with wheat, meat, and fish in the CM group and a positive correlation with meat and fish in the non-CM group (*p* < 0.001). In *panel D*, ASM (%) positively correlated with fat and meat intake in the CM group only (*p* < 0.001; Figure 1).

The number of participants with SO is presented in Table 4 by the two criteria; Sarcopenic Obesity^*a*:^ (*n* = 70) and Sarcopenic Obesity^*b*:^ (*n* = 155). Multivariate logistic regression was performed and presented between daily food sources and SO prevalence (Table 4). According to the tertiles of wheat intake (g/d), 2.1-fold increase in the risk of prevalence of SO (PR: 2.149, CIs: 1.134–4.071) was reported in the highest tertile (T3: 269.1 g/d) compared to that in the lowest tertile (T1: 20.0 g/d) in the CM group. Regarding meat intake (g/d), higher tertile (T2: 34.8 g/d, T3: 99.5 g/d) showed about 2-fold increase in the risk of prevalence of SO (PR: 1.932, CIs: 1.066–3.500) compared to the lowest tertile (T1: 9.2 g/d; Table 4).

**TABLE 4:** Adjusted prevalence ratios (PRs) in multivariate logistic regression between daily food sources and Sarcopenic Obesity.

	Sarcopenic Obesity*^a^*	Sarcopenic Obesity*^b^*
Daily food sources, (min.-max.)	(*n* = 70)	(*n* = 155)
	PRs (95% CIs)	
**Wheats*^c^* (g/day)**	ref. T1: 20.0 (0.0–51.7)	
T2: 101.2 (51.8–150.5)	0.784 (0.397–1.549)	0.994 (0.561–1.758)
T3: 269.1 (150.9–874.6)	1.793 (0.868–3.707)	**2.149 (1.134–4.071)**
P for trend	0.118	**0.016**
	PRs (95% CIs)	
**Meats (g/day)**	ref. T1: 9.2 (0.0–25.1)	
T2: 34.8 (25.2–45.0)	1.421 (0.709–2.850)	**2.554 (1.380–4.726)**
T3: 99.5 (45.2–717.4)	1.438 (0.712–2.904)	**1.932 (1.066–3.500)**
P for trend	0.323	**0.047**

*^a^ASM, kg/m^2^ by Asian Working Group for Sarcopenia (AWGS) 2019 criteria.*

*^b^ASM, % by Janssen’s formula [ASM/weight (kg) × 100 (%)].*

*^c^Wheats included wheat and other types of refined grains based extra food intakes for snacks; cereals, breads, ricecakes.*

*Multivariate logistic regression was used to estimate PRs (95% CIs) after adjusting covariates; age, sex, BMI, energy intake (kcal), smoking, alcohol consumption, physical activity, working hours, KM type, and CM pattern.*

*P for trend was tested across three levels (tertiles) of the food sources (g/day) by including the median score as a continuous measure in the regression model after adjusting all covariates. Bold letters represent a significant value.*

## Discussion

This study was performed to identify CM patterns in elderly Koreans living in a rural area, and examine the interrelationships between dietary factors, grip strength, body composition, and prevalence of SO with respect to CM pattern. The results indicated that overconsumption of wheat and meat, with imbalanced macronutrient intake, interacted with CM to produce significant effects on the risk of prevalence of SO by increasing muscle loss and/or body fat in elderly with CM.

### Sarcopenia, Obesity, and Cardiometabolic Diseases in Elderly

A recent large cohort study reported significantly higher prevalence of non-alcoholic fatty liver disease (NAFLD) in men with both T2DM and sarcopenia than in those without sarcopenia (39). A previous cross-sectional study found that coexistent sarcopenia and obesity had greater risks than did the single components or when associated with the multimorbidity prevalence in Korean adults (40). Elderly people are a vulnerable population as they are likely to have co-existing risk factors (41). In a Dutch Lifelines cohort study, the prevalence of SO increased with age, and SO participants with > 3 comorbidities had 2.7 times higher risk than those with no morbidities (42). Similarly, our results showed higher risk factors for body composition, cardiometabolic profiles, and prevalence of SO in the CM group than in the non-CM group. Multifactorial relationships have been hypothesized between SO and cardiometabolic diseases in elderly participants.

### Lifestyle Risk Factors of Sarcopenia, Obesity, Muscle Strength, Function, and Quality in Elderly With Cardiometabolic Multimorbidity

From an etiopathogenesis viewpoint, age- and sex-related physiological, hormonal, and body compositional changes, lifestyle factors such as diet, physical activity, and activities of daily living, and other unidentified factors might be mediators between CM and SO (7–9). Poor nutrition and physical inactivity are key contributors to sarcopenia and its risk (43, 44). A beneficial effect was reported in elderly sarcopenic adults who gained 1.7 kg of fat-free mass after participating in a resistance training program with dietary protein and vitamin D supplementation (45). However, excessive calorie intake from proteins might lead to altered body composition with higher body fat mass, lower muscle mass, and metabolic quality in older men with obesity (46). Consistently, elderly participants with CM patterns were more sedentary, more obese, had higher body fat mass, worsened cardiometabolic profiles, and high-risk sarcopenia or higher prevalence of SO than those without CM. Regarding the KM type, more participants in the CM group had Taeeum-type, with a predisposing metabolic risk (33, 34) than those in the non-CM group. Our ongoing cohort study showed a higher prevalence of pre-metabolic diseases (MetS), lower nutritional status, higher high-sensitivity C-reactive protein (hs-CRP) level, and lower vegetable consumption in Taeeum-type middle-aged Korean adults (33, 34). These results showed that individual personality or obesity-related physiological characteristics, which were associated to the KM type, might be negatively related to the occurrence of SO by uncontrolled diet and progressive reduction of physical activity in elderly participants with CM patterns.

### Inappropriate Macronutrient Intake Impacts Muscle Strength and Body Composition in Elderly With Cardiometabolic Multimorbidity

Different proportions of macronutrients in the diet have different effects on carbohydrate and fat storage in the human body (47). While overconsumption of carbohydrates promotes hepatic *de novo* “lipogenesis,” fat accumulation in adipose tissue promotes increased “lipolysis,” which are the main pathways that contribute to intrahepatic TG (48). Therefore, macronutrient composition in one’s diet acts as an important mediator. Repeated overconsumption of carbohydrate leads to development of MetS, which increases hepatic fat (47), serum TG levels, and reduces HDL-C (49). Consistently, a positive association between carbohydrate intake and the prevalence of MetS was reported in a previous Korean adult population-based study (17). The highest carbohydrate intake in men and refined grain intake in women were associated with elevated TG and blood glucose levels in combination with reduced HDL-C levels (17).

In aging muscle biology, dietary fatty acids (FAs) play an important role as activators by increasing levels of pro-inflammatory cytokines, which are implicated in muscle wasting (18). Saturated fatty acids (SFAs) influence IR and CVD by increasing serum low-density lipoprotein cholesterol (LDL) levels (50). Previous clinical research showed significant changes in higher fasting serum insulin levels after the consumption of an SFA-rich diet than of other unsaturated FA or carbohydrates (48). WHO reported that results from the regression analysis showed a more favorable effect on the serum lipoprotein profile of reducing dietary SFA intake by replacing with polyunsaturated or monounsaturated FA than with carbohydrate mixtures (51). A significantly higher inverse association between the omega-3 FA ratio and prevalence of SO has been reported in elderly women in the recent KNHANES data (52).

We found imbalanced macronutrient ratios, higher carbohydrate levels, and lower total fat intake in elderly participants compared to the KDRIs. Lower total protein adequacy (g/kg), excessive carbohydrate-induced muscle weakness, and fat intake related to a higher percentage of body fat mass in women were observed in the CM group. In addition, overconsumption of wheat and meat increased the risk of prevalence of SO about 2-folds in the CM group. We also identified protein adequacy as a contributor to improving muscle mass in elderly of both genders. These results suggest that elderly with CM make inappropriate food choices, such as the consumption of high-carbohydrate diets or high animal-fat with low protein foods. Therefore, making right food choices and adequate intake of carbohydrates and protein are recommended to improve IR and mitigate MetS, maintain muscle strength and quality, and prevent SO in elderly with CM.

### Nutritional Recommendation for Elderly With Cardiometabolic Multimorbidity to Improve Clinical and Nutritional Status

Saturated fatty acids are found mostly in animal products, as well as in some high-fat plant foods, such as palms, coconuts, avocados, olives, nuts, and seeds [Food Composition Table. 9.1 version. National Institute of Agricultural Sciences, Rural Development Administration of Korea (53)]. The American College of Cardiology/American Heart Association (ACC/AHA) guidelines recommends a reduced SFA intake of < 7% of total calorie intake to reduce the risk of CVDs (54). It is also recommended that SFAs should be replaced with polyunsaturated fatty acids (PUFAs) which lower TG (55) and cholesterol (56). Omega-6 and omega-3 PUFAs are the only essential dietary fats (57). The ideal ratio of omega-3/6 PUFAs is 1:1 to 1:4; however, an imbalance in the ratio of omega-3/omega-6 PUFAs ratio due to westernized diets, leads to increased synthesis of arachidonic acid from linoleic acid (55), accelerating the prevalence of atherosclerosis, chronic diseases such as obesity, and diabetes (56, 58).

Protein is a good dietary source for preserving muscle mass during weight loss (59). Research based on nationally representative data showed that optimal protein intake (at least 0.8 g/kg per day) reduced MetS risk (42%) and its components such as abdominal obesity (44%), lower HDL-C (47%), and elevated TG (45%) in 1,567 elderly participants (60). Another KNHANES report revealed protein-derived body composition changes and a higher prevalence of SO in 1,433 participants aged > 60 years (19).

Plant-based proteins, such as soy products, have high-quality sustainable proteins for optimal muscle protein synthesis, to prevent muscle loss, and manage weight by reducing hunger (61). Nuts, seeds, and legumes are other beneficial dietary sources of protein, magnesium, and fiber. However, they should be taken in small portions without added salt or sugar (54). Mediterranean diets (62) and dietary approaches to stop hypertension (DASH) (63) diets are well-known nutritional therapies high in vegetables and fruits, low-fat dairy products, fish, and legumes and low in red-meat, SFA, and sugars. Adherence to these healthy dietary patterns has favorable effects on CVD mortality and improves cardiometabolic markers (64) and intrahepatic fat (65) in older adults. A plant-based diet is also encouraged as an ideal diet for the elderly population as it lowers the risk of cardiovascular disease, provides protective effects through multiple beneficial nutrients, and includes a wide range of antioxidants (56).

In Asian or Korean culture, carbohydrate-based diet (rice-based meals) with various vegetables and low-fat protein sources (fish and seafood, legumes, and bean/tofu-based side dishes) was the usual diet of those who lived in rural/coastal areas for their whole life. Both CM and non-CM participants adhere to traditional rural lifestyle, which consists of the consumption of high-carbohydrate diets and spending most of their time working. However, age-related body composition changes, obesity-related chronic multi-disease status, and imbalanced macronutrients might reduce protein synthesis in chronic catabolic states, leading to worsening muscle strength and quality in Korean elderly people with CM.

The WHO recommends an intake of > 400 g/d of non-starch fruits and vegetables to prevent chronic diseases (66). Replacing plant-based protein intake and n-3 FAs (vegetable oils, soybean, rapeseed oil, walnuts, and fish oils) might enhance muscle mass and strength in women according to individual differences in age, sex, muscle capability and function, exercise, and menopause. Lastly, active daily living and physical activity could improve protein metabolism and counteract anabolic stimulus in muscle loss in the elderly population with CM.

### Strength and Limitation

To the best of our knowledge, this study is the first study to investigate the relationship between dietary factors, grip strength, body composition, and prevalence of SO in Korean rural residents according to their CM pattern. We employed multiple representative definition criteria for the diagnosis of SO by adapting the AWGS 2019 criteria and Janssen’s formula.

This study has some limitations. Being a cross-sectional study, it was difficult to make causal inferences due to limited evidence. Second, we did not consider other chronic diseases, multimorbidity pattern effects, or periods of disease in the present study. Lastly, we evaluated only residents of a rural area in Korea; therefore, careful insights are required when interpreting the present data for the sake of generalizability.

## Conclusion

This study highlights that overconsumption of wheat and meat negatively impacts the prevalence of SO in Korean elderly with CM. Furthermore, nutritional management is strongly required to improve cardiometabolic profiles and macronutrient imbalance, protein adequacy, and food choices (avoiding high-carbohydrates/fat-based snacks or meals). A patient-centered integrated multisectoral team-based approach is required to manage systematic guidelines for diet and health in older adults with multiple chronic diseases.

## Data Availability Statement

The datasets are not available due to confidentiality and security of ethical issues; further inquiries can be directed to the corresponding authors.

## Ethics Statement

The studies involving human participants were reviewed and approved by the Korean Institute of Oriental Medicine Ethics Committee (No. I-1401/001-001-01) and the Ethics Committee of the Institute of Medicine at the Seoul National University (IRB No. 1310-060-528). The patients/participants provided their written informed consent to participate in this study.

## Author Contributions

JK: conceptualization, analysis and interpretation of data, and original manuscript. KJ: data curation. YB and SL: review and editing. SL: funding acquisition and project administration. All authors contributed to the article and approved the submitted version.

## Conflict of Interest

The authors declare that the research was conducted in the absence of any commercial or financial relationships that could be construed as a potential conflict of interest.

## Publisher’s Note

All claims expressed in this article are solely those of the authors and do not necessarily represent those of their affiliated organizations, or those of the publisher, the editors and the reviewers. Any product that may be evaluated in this article, or claim that may be made by its manufacturer, is not guaranteed or endorsed by the publisher.

## Abbreviations

AWGS: Asian Working Group for Sarcopenia
ASM: appendicular skeletal muscle mass
BIA: bioelectrical impedance analysis
BMI: body mass index
BP: blood pressure
CI: confidence interval
CM: cardiometabolic multimorbidity
CVD: cardiovascular disease
FA: fatty acid
HDL: high-density lipoprotein
IR: insulin resistance
KDRIs: Korean Dietary Reference Intakes
KM: Korean Medicine
KNHANES: Korean National Health and Nutrition Examinations Survey
MetS: metabolic diseases
NAFLD: non-alcoholic fatty liver disease
OR: odds ratio
PR: prevalence ratio
SD: standard deviation
SFA: saturated fatty acid
SO: sarcopenic obesity
T2DM: type 2 diabetes
TG: triglycerides.
