# Supplementary material for: Association of Dietary Factors With Grip Strength, Body Fat, and Prevalence of Sarcopenic Obesity in Rural Korean Elderly With Cardiometabolic Multimorbidity
Source: Front Nutr. 2022 Jul 14;9:910481. doi: 10.3389/fnut.2022.910481 (PMC9329691; doi:10.3389/fnut.2022.910481)
Supplement: Supplementary file 1 [file Table_1.docx]

**Supplementary Table 1: Orthogonal Quartimax Rotated Loadings of Four Factors of Multimorbidity patterns Based on Tetrachoric Correlation Matrix**

| **Condition** | Factor 1 | Factor 2 | Factor 3 | Factor 4 |
| --- | --- | --- | --- | --- |
| Stroke | -0.03 | 0.13 | -0.17 | 0.20 |
| Transient ischemic attack | 0.10 | 0.10 | -0.34 | 0.23 |
| Angina | 0.04 | -0.01 | -0.04 | **0.42** |
| Hypertension | -0.07 | 0.01 | -0.02 | **0.46** |
| Dyslipidemia | 0.00 | -0.02 | 0.02 | **0.58** |
| Pulmonary tuberculosis | **0.56** | -0.01 | -0.03 | 0.00 |
| Thyroid disorder | 0.00 | 0.28 | -0.12 | 0.22 |
| Chronic gastritis | 0.19 | **0.34** | 0.12 | -0.09 |
| Ulcer | 0.17 | 0.06 | 0.02 | -0.06 |
| Diabetes | -0.10 | -0.04 | 0.10 | **0.65** |
| Intestinal polyp | 0.25 | 0.28 | 0.12 | -0.07 |
| Acute hepatic disease | 0.27 | 0.04 | **0.43** | -0.01 |
| Fatty liver | 0.21 | -0.09 | **0.43** | 0.20 |
| Chronic hepatitis | **0.39** | -0.01 | **0.30** | -0.02 |
| Cholelithiasis | -0.03 | 0.08 | 0.25 | 0.14 |
| Chronic bronchitis | **0.67** | -0.09 | -0.08 | 0.04 |
| Asthma | **0.60** | 0.11 | -0.04 | 0.03 |
| Allergy | **0.47** | 0.15 | -0.39 | 0.01 |
| Arthritis | 0.01 | **0.55** | -0.02 | 0.00 |
| Cystitis | 0.07 | **0.38** | 0.22 | -0.01 |
| Cataract | 0.01 | **0.52** | 0.10 | 0.14 |
| Glaucoma | 0.04 | 0.10 | **0.33** | 0.05 |
| Depression | 0.11 | 0.30 | **0.45** | -0.06 |
| Parkinson’s disease | -0.03 | -0.09 | -0.06 | -0.11 |
| Osteoporosis | 0.02 | **0.63** | -0.11 | 0.04 |
| Prostatic hyperplasia | 0.18 | -0.26 | 0.17 | 0.11 |
| Cancer1 | -0.11 | -0.06 | **0.26** | -0.05 |
| Cancer2 | -0.08 | 0.04 | **0.28** | -0.04 |
| Cancer3 | 0.00 | 0.00 | 0.00 | 0.00 |

Number of factors was determined by retaining factors with an eigenvalue greater than one.

Bolded values are factor loadings with a magnitude greater than 0.3.
